# Magnetic resonance imaging for the diagnosis of malignant mixed Mullerian tumor of ovary: Two case reports

**DOI:** 10.1097/MD.0000000000036569

**Published:** 2023-12-15

**Authors:** Xiaofeng Jin, Ping Zhu, Shufeng Fan, Jingru Dai

**Affiliations:** a Department of Radiology, The Second Affiliated Hospital of Zhejiang Chinese Medical University, Hang Zhou, Zhejiang, China.

**Keywords:** magnetic resonance imaging, malignant mixed Mullerian tumor, ovarian tumor

## Abstract

**Rationale:**

Malignant mixed Mullerian tumor (MMMT) is also known as carcinosarcoma, mostly occurring in the uterus, and occurred in ovary is very rare. The disease is highly aggressive. Two cases of MMMT of ovary and their imaging characteristics were collected in our study.

**Patient concerns:**

A 77-year-old and an 80-year-old woman were admitted to the obstetrics and gynecology department of our hospital on June 22, 2019, and December 10, 2019, respectively. The first patient presented with abdominal distension with poor appetite without obvious triggers. Another patient had been menopausal for 18 years and presented with vaginal bleeding with dull pain in the left lower abdomen without obvious cause.

**Diagnoses:**

Both patients underwent pelvic magnetic resonance imaging plain and enhanced scan after admission, which indicated pelvic mass. Postoperative pathology confirmed MMMT in the adnexal region.

**Interventions:**

Both patients underwent total hysterectomy and bilateral adnexectomy.

**Outcomes:**

Postoperatively, the first patient developed complications such as renal failure and gastrointestinal bleeding and was sometimes unconscious. Symptomatic treatment was not effective, and the patient died about 1 month after discharge. The other patient recovered well after surgery, and imaging examinations confirmed no evidence of regrowth of the tumor during an average 36-month follow-up.

**Lessons:**

The disease is highly malignant and progresses rapidly. The elevation of CA125 should be taken seriously. The imaging findings of MMMT has certain characteristics. Multi-sequence magnetic resonance imaging may help to distinguish this disease from other pelvic tumors. Once found, surgical treatment is needed as soon as possible, followed by postoperative adjuvant radiotherapy and chemotherapy.

## 1. Introduction

Malignant mixed Mullerian tumor (MMMT) of the ovary is a kind of tumor that is composed of malignant mesenchymal components and malignant epithelial components.^[1,2]^ It contains both sarcoma and carcinoma components, also known as ovarian carcinosarcoma, with high aggression. This disease is extremely rare; malignant mixed Mullerian of the adnexal region account for only 0.1% to 0.5% of the malignant tumors in the genital tract, and malignant mixed Mullerian of the ovary account for 1% to 4% of all ovarian tumors.^[1,3]^ The clinical symptoms are atypical, and the common clinical symptoms are abdominal mass and abdominal distension with or without vaginal bleeding. The early diagnosis of this disease is difficult, mostly confirmed by surgical pathology and the prognosis is poor. Therefore, preoperative diagnosis is very important. There are few imaging reports about MMMT of the ovary.^[4,5]^ Multi-sequence magnetic resonance imaging (MRI) can show the signals of different pathological components, which may be an effective method for preoperative diagnosis of malignant Mullerian mixed tumor of ovary. In this study, 2 patients of malignant Mullerian mixed tumor of ovary were reported. The diagnosis was made by multi-sequence MRI, which were surgically resected, and the specimens were confirmed by pathology.

This study has obtained the informed consent of the patient (or her immediate family member).

## 2. Case report

### 2.1. Case 1

#### 2.1.1. Medical history.

A 77-year-old female patient presented with abdominal distension and poor appetite >10 days ago without obvious cause. She had hypertension for >10 years and diabetes for >20 years. She took antihypertensive drugs regularly and injected insulin early and late every day. She did not monitor blood pressure and blood sugar level at ordinary times.

#### 2.1.2. Auxiliary examination results.

**Figure 1.**
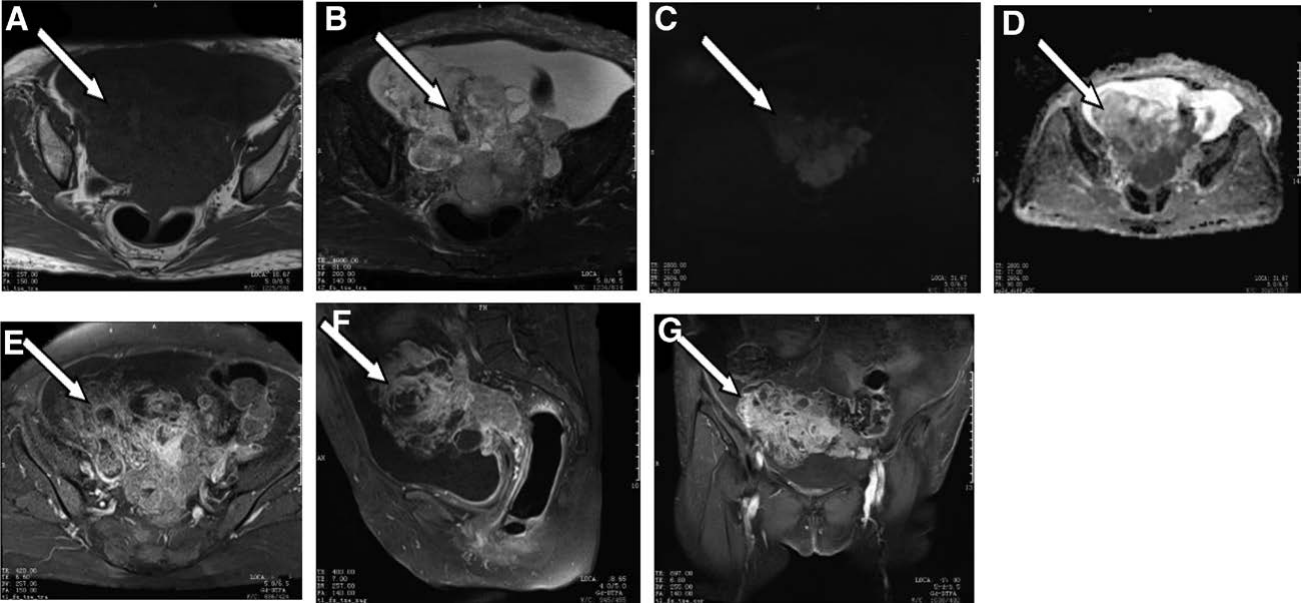
Case 1. Malignant mixed Mullerian tumor of the right adnexal region. (A) T1WI. (B) FS-T2WI. (C) DWI. (D) ADC. (E–G) Contrast enhancement. ADC = apparent diffusion coefficient, DWI = diffusion-weighted imaging, T1WI = T1-weighted image, T2WI = T2-weighted image.

After admission, the “7 tumor indicators” showed CA125 1252.8 U/mL, CA15-3 197.6 U/mL, CYFRA21-1 33.37 ng/mL. Routine blood tests performed at our hospital on June 22, 2019, revealed the following: neutrophil percentage, 75.2%; lymphocyte percentage, 16.9%. Blood biochemistry results revealed the following levels: sodium, 135.4 mmol/L; chloride, 98.9 mmol/L; albumin, 32.4 g/L; creatinine, 83.4 µmol/L; glucose, 1.87 mmol/L. The coagulation and fibrinolytic tests revealed that D-dimer was 3.96 mg/L and fibrinogen was 5.13 g/L. Routine urine tests revealed normal results. The color Doppler ultrasound of the uterus and adnexa showed cystic-solid echo masses in the pelvic cavity. *Pelvic effusion*. Pelvic MRI plain scan and enhancement showed that the abnormal signal mass in the pelvic cavity was about 13.7 × 10.1 × 10.9 cm in size, and the signal was uneven. T1-weighted image (T1WI) showed equal and low signal, T2-weighted image (T2WI) showed equal and high signal, diffusion-weighted imaging (DWI) showed high signal, and apparent diffusion coefficient (ADC) value was significantly reduced. After enhancement, the lesions showed obvious heterogeneous enhancement in the arterial phase, and the degree of enhancement decreased in the delayed phase. The adjacent uterine body and fundus showed obvious enhancement. The surrounding pelvic soft tissues were shown as patchy and grid-like FS-T2WI high-signal images (Fig. 1). The diagnosis was pelvic mass and considered to be a malignant tumor of adnexal origin with invasion of the uterus. The surrounding soft tissue was edematous. Massive fluid accumulation in the pelvic cavity.

#### 2.1.3. Surgical management and histopathology.

Subsequently, the patient underwent total hysterectomy + bilateral adnexectomy + appendectomy + omentum resection + pelvic mass resection under general anesthesia. Postoperative pathology showed a MMMT of the right adnexal region (uterus + double adnexa). Tumor invasion was found in the soft tissues around the bilateral adnexa, the prevesical peritoneum, and the omentum. Tumor invasion or metastasis was found in the abdominal wall. There was no tumor invasion of the appendix. The cervical basal margin was negative. Immunohistochemistry showed ER (glandular epithelium 60%+), PR (glandular epithelium and stroma, 80%+), Vim+, EMA+, CK7+, CA125+, CK20−, SMA−, S-100−, P53+, Ki67 60%+.

#### 2.1.4. Follow-up.

After the operation, the patient was in poor mental condition, and her consciousness was sometimes awake and sometimes blurred. Symptoms such as renal failure and gastrointestinal bleeding occurred and was given symptomatic support treatment, but the effect was not good. After 19 days of hospitalization, the patient and her family decided to give up treatment and asked to be discharged. We regret to learn that the patient died about 1 month after discharge.

### 2.2. Case 2

#### 2.2.1. Medical history.

An 80-year-old female patient presented with postmenopausal vaginal bleeding for 1 month. One month ago, the patient developed vaginal bleeding without obvious inductance, with a small amount and light red color, and occasionally felt a dull pain in the left lower abdomen. She had a history of hypertension for >10 years. She took medication regularly and reported that her blood pressure could be controlled.

#### 2.2.2. Auxiliary examination results.

**Figure 2.**
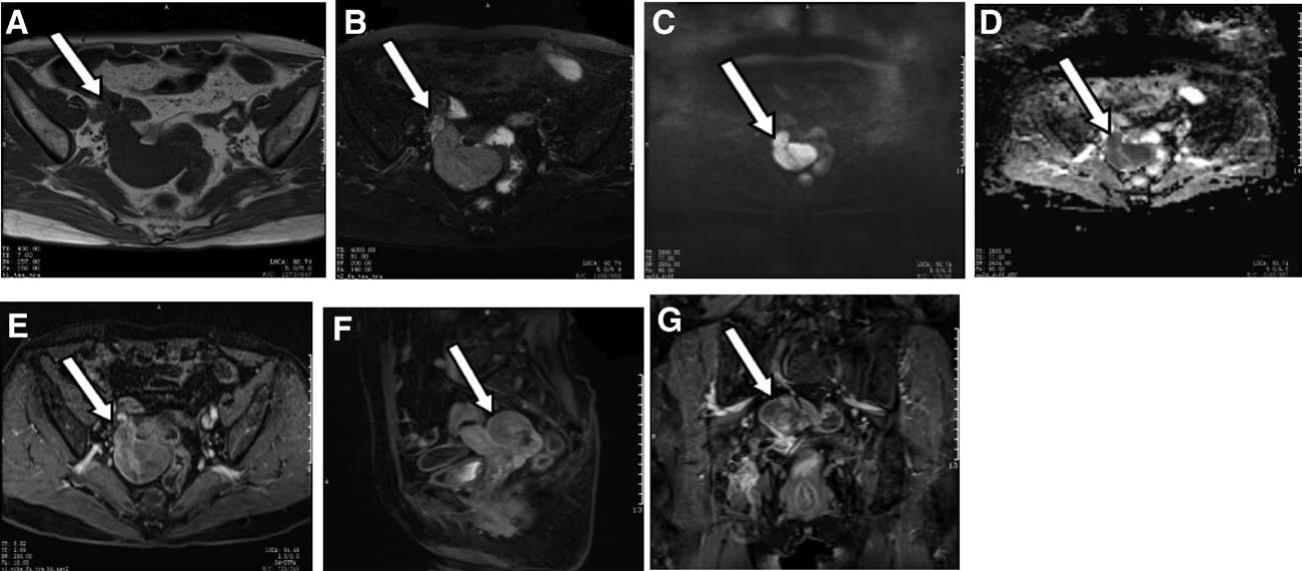
Case 2. Malignant mixed Mullerian tumor of the right fallopian tube. (A) T1WI. (B) FS-T2WI. (C) DWI. (D) ADC. (E–G). Contrast enhancement. ADC = apparent diffusion coefficient, DWI = diffusion-weighted imaging, T1WI = T1-weighted image, T2WI = T2-weighted image.

The “7 tumor indicators” showed AFP 9.7 ng/mL, CA125 55.6 U/mL, CYFRA21-1 2.64 ng/mL. Routine blood test results on December 10, 2019 are as follows: monocytes percentage, 11.5%; and ultrasensitive C-reactive protein level, 8.5 mg/L. The color Doppler ultrasound of the uterus and adnexa showed hypoechoic mass in right posterior of uterus. Pelvic MRI plain scan and enhancement showed that there were multiple abnormal signals in the pelvic cavity, the larger one was about 5.7 × 3.5 ×  5.0 cm. T1WI showed equal signal, T2WI showed mainly high signal with scattered stripe low signal, DWI showed high signal, and ADC value was decreased. The lesions were closely related to the right adnexal area. The enhancement was heterogeneous in arterial phase and reduced in delayed phase (Fig. 2). The diagnosis was pelvic mass and adnexal origin or sex cord-interstitial origin malignancy was considered.

#### 2.2.3. Surgical management and histopathology.

Then, the patient underwent transabdominal bilateral adnexectomy + total hysterectomy + pelvic lymph node dissection + para-aortic lymphadenectomy + omentectomy + peritoneal biopsy under general anesthesia. Postoperative pathology showed a MMMT (carcinosarcoma, tumor size 7*6*4 cm) of the right fallopian tube with necrosis and invasion of the serous layer of the tube wall. There was no cancer metastasis in the lymph nodes. Immunohistochemistry showed P16+, Vim+, P53+, PR 70%+, (CK7, EMA, SMA) part +, S-100+, ER−, CK20−, CA125−, Ki67 70%+.

#### 2.2.4. Follow-up.

The patient recovered well after surgery. After 17 days of hospitalization, the patient and her family requested to be discharged. At a mean follow-up of 36 months, the patient had no further episodes of symptoms and computed tomography/MRI showed no evidence of tumor regrowth.

## 3. Discussion

MMMTs of the ovary are rare, with highly malignant and early metastasis. Compared with ovarian cancer, it has a lower chemotherapy response rate and a worse prognosis.^[6]^ The etiology of the disease is unclear. It has been reported that long-term use of tamoxifen and pelvic irradiation may be risk factors, while oral contraceptives are protective factors.^[7]^ The clinical symptoms are nonspecific, usually with abdominal pain, vaginal bleeding, and abdominal or pelvic mass.^[8]^ The treatment method is surgery and postoperative chemotherapy.^[9]^ At present, MMMT is diagnosed by postoperative pathology. In this study, the clinical and MRI findings of 2 patients with this disease were studied to determine the possible imaging features. It may help to distinguish this disease from other pelvic tumors.

In the first case, the tumor signal was obviously heterogeneous, which was considered as cystic degeneration or cystic components, hemorrhage, and necrosis in the lesion. It has been reported in the literature that the cystic component may be related to the secretion of mucus or serous fluid by adenocarcinoma.^[10]^ Intracystinal hemorrhage is caused by the high degree of malignancy of the tumor, rapid growth, and local blood supply disorder or relative insufficiency of blood supply.^[11]^ The second patient presented with pelvic solid mass, which showed isointense signal on T1WI, high signal on T2WI, high signal on DWI, and low ADC value, showing the imaging characteristics of malignant tumor. Scattered strip-like low signal within the high signal on T2WI were considered to be empty flow vessel. Some scholars believe that the empty flow vessel is the characteristic image sign of MMMT.^[12]^ Both cases showed obvious heterogeneous enhancement in the arterial phase and decreased enhancement in the delayed phase, which may be due to the mixed epithelial and mesenchymal tissue in the tumor. In terms of tumor markers, both patients showed a significant increase in CA125, which was considered to be MMMT of the ovary with epithelial origin.^[13]^

In differential diagnosis, MMMT needs to be differentiated from ovarian epithelial tumors, germ cell tumors, sex cord-stromal tumors, and metastatic tumors. Epithelial ovarian cancer with predominantly solid and mixed cystic and solid components is usually accompanied by massive ascites, whereas MMMT rarely presents with ascites. Neither patient in this study had ascites. Endometrioid carcinoma may present with solid and cystic features similar to MMMT, but usually with a history of endometriosis. The imaging features of dysgerminoma in germ cell tumors are lobulated masses with obvious fibrous septa. Dysgerminoma tends to occur in adolescent and childbearing-age women, and the enhancement is uniform. MMMT usually occurs in postmenopausal women. Granulosa cell tumors in sex cord-stromal tumors are mostly multilocular cystic masses with irregular morphology. The cystic wall and solid components showed obvious enhancement on contrast-enhanced scan, which was similar to MMMT. However, its estrogen level was significantly higher than normal, which could be distinguished from MMMT.

### 3.1. Limitation

First, clinical reports on MMMT of ovary are relatively rare. Our report is based on literature of 2 clinical cases, and more relevant clinical cases are needed to further study the imaging features. Second, MMMT’s clinical symptoms has no specificity and more likely to be misdiagnosed, which delays the treatment and diagnosis of this disease.

## 4. Conclusion

We described 2 extremely rare cases of giant MMMTs of ovary. Their MRI features have certain characteristics: The lesions showed equal or high signal on T1WI, high or mixed high signal on T2WI, high signal on DWI, and decreased ADC value. The arterial phase of enhanced scan showed obvious heterogeneous enhancement, and the degree of enhancement decreased in the delayed phase, showing the image characteristics of malignant tumors. The possibility of malignant Mullerian mixed tumor of ovary should be considered in differential diagnosis when cystic degeneration, necrosis, and hemorrhage occur in the tumor, especially when there are signs of empty blood vessel. Understanding the continual updating of radiological and pathomorphological features of those patients is of great significance for their diagnostic and treatment. Our report may help to deepen the understanding of this rare clinical entity.

## Author contributions

**Conceptualization:** Xiaofeng Jin, Jingru Dai.

**Data curation:** Xiaofeng Jin.

**Funding acquisition:** Shufeng Fan.

**Methodology:** Xiaofeng Jin, Jingru Dai, Ping Zhu.

**Resources:** Xiaofeng Jin.

**Software:** Jingru Dai.

**Writing – original draft:** Xiaofeng Jin, Jingru Dai.

**Writing – review & editing:** Ping Zhu, Shufeng Fan.
